# Numerical Simulation of the Effect of Process Parameters on Pass Filling Degree in F-Section Steel Finishing Rolling Process

**DOI:** 10.3390/ma19102058

**Published:** 2026-05-14

**Authors:** Huiyuan Duan, Li Jin, Ruxin Xiao, Yang Gao, Xu Li, Jingguo Ding

**Affiliations:** State Key Laboratory of Digital Steel, Northeastern University, Shenyang 110819, China; 15713542988@163.com (H.D.); 2310166@stu.neu.edu.cn (L.J.); 2300701@stu.neu.edu.cn (R.X.); gaoyang@ral.neu.edu.cn (Y.G.); lixu@ral.neu.edu.cn (X.L.)

**Keywords:** F-section steel, pass filling degree, simulation, friction coefficient, tension configurations, rolling temperature, web reduction

## Abstract

Due to the asymmetry of pass profiles, F-section steel is prone to defects such as overfilling, underfilling, and twisting during production, which significantly deteriorates the dimensional accuracy, mechanical properties, and surface quality of products. To mitigate the occurrence of such defects, this study established a thermo-mechanical coupled three-dimensional finite element model for the finishing rolling process of F-section steel using ABAQUS 2022 incorporating the actual operating conditions of the steel plant’s production line. By analyzing the stress–strain fields of each pass, it was found that the maximum deformation of the rolled piece is concentrated at the junctions of the inner leg with the flange, the inner leg with the web, and the outer leg with the web. Additionally, underfilling was observed at the legs and flanges of the pass in each rolling sequence. Based on these findings, an in-depth analysis was conducted on the effects of friction coefficient, tension configuration, rolling temperature, and web reduction on pass filling degree. Conditions of low friction, small reduction, and high temperature facilitate the smooth filling of metal in the leg cavity; in contrast, conditions of high friction, large reduction, and low temperature promote the filling of surface metal and an increase in spread. Maintaining a low-tension state is a common favorable condition for improving the pass filling degree of both the legs and the surface. When the friction coefficient is 0.2, tension is 0, rolling temperature is 1040 °C, and web reduction is 4 mm, the pass filling degrees of the inner and outer legs reach their maximum values of 99.88% and 99.16%, respectively. When the friction coefficient is 0.4, tension is 0, rolling temperature is 1010 °C, and web reduction is 4 mm, the pass filling degrees of the upper and lower surfaces are maximized, reaching 98.95% and 98.22%, respectively. These findings provide data support and theoretical guidance for addressing defects encountered in F-section steel production.

## 1. Introduction

Medium–low speed maglev track panels consist of F-section steel guide rails and H-section steel sleepers. As the core load-bearing and guiding component of the maglev track system, the F-section steel guide rail directly affects the operational stability, safety, and economic efficiency of maglev trains [1,2,3]. F-section steel track technology, characterized by environmental friendliness, safety, and strong climbing capacity, has been identified as a key direction of national strategic emerging industries [4,5]. Compared with traditional cold-formed technology, its hot-rolling process offers superior material strength and longer service life. With the rapid advancement of urban rail transit, the demand for F-section steel guide rails has been steadily rising [6,7].

Due to the cross-sectional asymmetry of F-section steel, its rolling centerline does not align with the roll centerline, posing significant challenges to roll matching. During rolling, defects such as twisting, head lifting, or head bowing are prone to occur, requiring repeated iterations of pass modification, trial rolling, re-modification, and re-trial rolling until the final trial rolling is successful. In actual production, there also exist intractable issues that are difficult to quantitatively predict via empirical judgment, including overfilling, underfilling, folding, and leg elongation in typical passes. These problems result in substantial resource waste, prolonged product trial cycles, and increased R&D costs; in severe cases, they may even delay delivery schedules [8,9]. Thus, leveraging numerical simulation technology to analyze the complex deformation mechanisms during the hot rolling of F-section steel holds significant importance for controlling product dimensional accuracy, reducing surface defects, and cutting production costs.

In recent years, finite element numerical simulation has become the core method for deciphering complex rolling mechanisms and optimizing process parameters. Ojeda-López et al. [10] systematically reviewed numerical simulation tools in rolling processes, emphasizing their irreplaceability in process development. Ervasti and Ståhlberg [11] employed numerical simulation techniques to investigate the heterogeneous influence mechanisms of roll radius, reduction ratio, and inclusion morphology on metal flow during the single-pass hot rolling of steel billets. They identified that local stress concentration is the fundamental mechanical cause inducing the propagation of microcracks and pores within the rolled piece. Kim and Im [12] pioneered the 3D finite element analysis of non-isothermal shape rolling, laying the foundation for thermo-mechanical simulations of complex cross-sections. Tajik et al. [13] integrated numerical simulation with experimental validation to reveal that uneven strain distribution along the cross-sectional thickness direction and asymmetric stress paths are the fundamental mechanical origins of torsional deformation. For highly asymmetric L-section profiles, Oh et al. [14] employed finite element software to investigate the evolution of process-induced defects during ring rolling, uncovering the micromechanical formation mechanism of “folding” at flange corners and “local underfilling” at the bottom—defects driven by asymmetric metal flow velocity differences.

Focusing on a novel inclined elliptical–circular pass system, Nogayev et al. [15] explored the three-dimensional deformation characteristics of complex asymmetric passes, as well as the metal flow behavior and shear strain distribution within inclined passes. Based on DEFORM-3D software, they established a multi-objective optimization model targeting maximum effective strain and minimum rolling force. Graça and Vincze [16] provided a systematic review of FEM methods for asymmetric rolling processes. Akkaş et al. [17] focused on bulb flat steel for shipbuilding, successfully revealing the deformation laws and optimizing the pass design through computer-aided 3D analysis. Pan et al. [18] integrated experimental methods with three-dimensional finite element technology to investigate the rheological evolution mechanism of surface defects during heavy rail hot rolling, successfully addressing the limitation that traditional approaches struggle to achieve full-process microscopic tracking of defect closure and distortion. In cold roll forming, Wang et al. [19] identified longitudinal strain discrepancies as the intrinsic cause of “twist” defects in asymmetric automotive side beams. Extending this to highly asymmetric Z-section steel [20], the team clarified that stress imbalances from uneven force distribution drive twist distortion, thereby developing a 3D-FE optimization method to minimize this deformation.

At present, in the field of special-section steel rolling, while numerous scholars have achieved substantial progress in research on cross-sectional metal flow and defect prediction, studies on pass filling degree remain relatively scarce and superficial, with no quantitative analysis of this parameter having been conducted to date. Additionally, relevant research on the effects of tension mechanisms and rolling temperatures on pass filling degree is still insufficient. This paper elaborates in detail on the influence laws of tension mechanisms and rolling temperatures on the pass filling degree and width variation of F-section steel, thereby filling the research gap regarding the impact of the aforementioned factors on the forming process of special-section steel.

## 2. FEM Model Establishments

The rolling model for the finishing stage of F-section steel was established by ABAQUS 2022 finite element simulation. As an asymmetric special-section steel, the pass filling degree of the workpiece in each rolling pass not only reflects the metal flow distribution but also governs the dimensional accuracy of the final product. By analyzing the deformation results of the rolled piece and pass filling degree during the stable rolling stage, it can effectively provide theoretical guidance for on-site rolling production.

### 2.1. Finite Element Modeling Process

**Figure 1:**
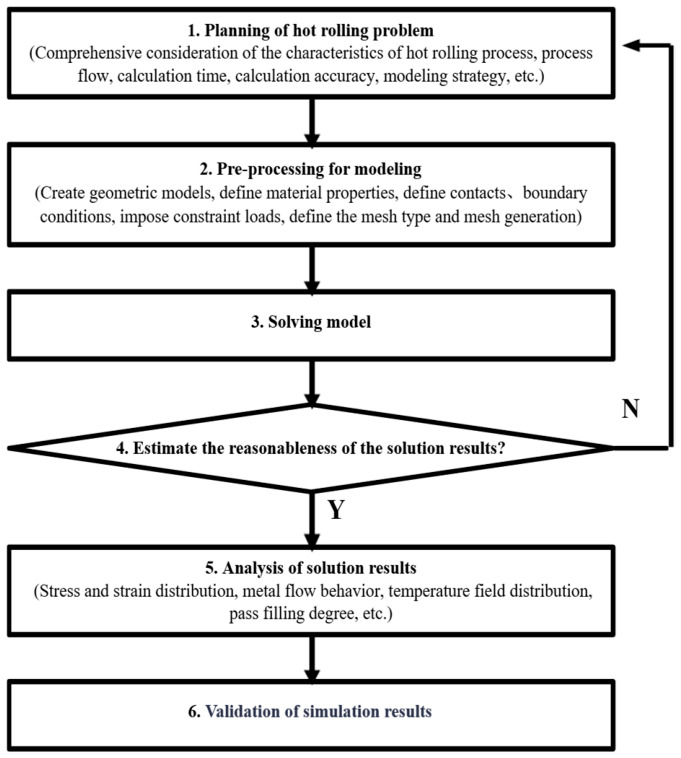
ABAQUS analysis flow chart of hot-rolling problem.

The ABAQUS finite element modeling and analysis process can be shown in Figure 1. A single-pass thermo-mechanical coupling numerical simulation is performed to verify the applicability of boundary conditions and validate the reasonableness of the solution results. If the results are reasonable, subsequent multi-pass thermo-mechanical coupling numerical simulations are carried out; if not, the model is revised and the solution process is repeated.

During the discretization of the geometric model into a finite element framework, a denser mesh typically yields higher precision at the expense of increased computational cost. Therefore, conducting a mesh sensitivity analysis—evaluating various element sizes and topological strategies—is essential to determine an optimal configuration that balances robust simulation accuracy with computational efficiency.

Given that finite element simulations must be executed sequentially for each individual operating condition, calibrating the model’s precision is highly time consuming. Furthermore, the developed model generates a massive volume of computational data. Evaluating key parameters—such as the heights of the inner and outer legs, the widths of the upper and lower surfaces, the spread distribution of the rolled piece and the rolling force—requires extracting coordinate and computational data from element nodes for post-processing. To significantly enhance the efficiency of this workflow, automated programming scripts were utilized to directly retrieve the finite element nodal data.

### 2.2. Determination of Material Model

The accuracy of material parameters directly dictates the reliability of calculation results in numerical simulations. In this study, the rolls are modeled as rigid bodies and the rolled piece is made of Q235B. The chemical composition of Q235B steel is as follows: carbon (C) content of 0.18%, silicon (Si) content of 0.15%, manganese (Mn) content of 0.40%, phosphorus (P) content no more than 0.025%, and sulfur (S) content no more than 0.012%. Accounting for the effects of temperature and strain rate on material parameters, JMatPro-computed thermophysical and mechanical properties are imported into the finite element model as input data. The specific parameters are presented in Table 1.

The effect of deformation rate on deformation resistance is primarily governed by the contradictory interplay between hardening and softening processes occurring within the metal during plastic deformation. An increase in deformation rate elevates the heat generation rate per unit time, which facilitates softening and thus reduces deformation resistance. Conversely, a higher deformation rate shortens the deformation duration, leading to insufficient initiation and propagation of dislocation motion during plastic deformation, thereby increasing deformation resistance.

**Figure 2:**
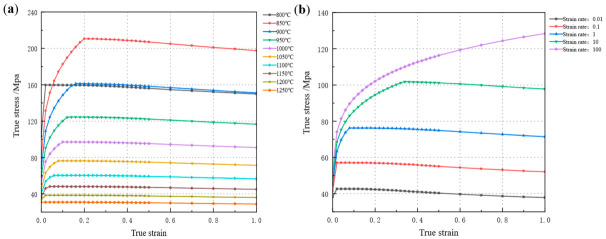
The stress–strain curve diagrams at different temperatures and strain rates. (**a**) At a fixed strain rate of 1 s^−1^ with varying temperatures, (**b**) at a fixed temperature of 1050 °C with varying strain rates.

The temperature of the rolled workpiece upon furnace exit is 1200 °C. After the breakdown rolling stage, the temperature is approximately 1050 °C. Prior to the initiation of the finishing rolling stage, the temperature is maintained at no less than 1040 °C. Following the completion of finishing rolling, the temperature is around 950 °C. In accordance with the temperature specifications for each stage of the rolling process, test temperature measurement points are set at 50 °C intervals ranging from 800 °C to 1250 °C. To accurately investigate the effect of strain rate on deformation resistance, the variation range of instantaneous strain rate must be considered. Accordingly, five strain rates (0.01 s^−1^, 0.1 s^−1^, 1 s^−1^, 10 s^−1^, 100 s^−1^) are designated for each temperature point. The true stress–strain curves obtained under varying temperatures and strain rates are presented in Figure 2.

### 2.3. Definition of Friction Model and Thermal Boundary Conditions

The friction model adopted in this study is the Coulomb friction model, and it is believed that the friction coefficient is related to temperature and the relative speed of the contact surface. When *T* > 700 °C and *V* < 5 m/s, the friction coefficient between the rolls and the rolled piece is as shown in Formula (1), *T* represents the temperature of the rolled piece, with the unit being °C; *V* represents the speed of the rolls relative to the rolled piece, with the unit being (m/s).

(1) μ=1.05−0.0005T−0.056V

During the hot-rolling process, the speed of the rolled piece gradually increases, while the surface temperature of the rolled piece gradually decreases. Therefore, the friction coefficient fluctuates within a certain range. Drawing on the relevant literature, the friction coefficient typically ranges from 0.2 to 0.5, and a constant value of 0.3 is commonly employed in most finite element simulations [21,22,23,24,25]. In subsequent work, to investigate the effect of friction coefficient variations on the pass filling degree of the rolled piece, this study quantitatively selects values of 0.2, 0.3, and 0.4 for simulation analysis.

The specific parameters of the thermal boundary conditions during the hot-rolling process of F-section steel are shown in Table 2. In the load module of the boundary conditions, the roll temperature is specified as 200 °C, while the initial temperature of the rolled workpiece is set to 1200 °C. Within the interaction module, three thermal exchange mechanisms are defined: convective heat transfer between the rolled workpiece and ambient air, radiative heat dissipation, and contact heat conduction at the roll–workpiece interface. The convective heat transfer coefficient is assigned a value of 0.065 (mW/(mm^2^·°C)), the surface emissivity is set to 0.8, and the ambient temperature is fixed at 20 °C. The frictional heat generation coefficient is defined in the contact property settings as 0.5, whereas the plastic work heat generation coefficient is specified via the inelastic heat fraction parameter in the material properties, typically adopting a value of 0.9.

In order to accurately analyze the heat transfer process between the rolled piece and the rolls, this paper referred to relevant literature data and conducted multiple finite element simulation experiments to modify the contact heat transfer coefficient. The modified contact heat transfer coefficient is shown in Formula (2), *K* represents the contact heat transfer coefficient, with the unit being (mW/(mm.°C)). *d* represents the distance between the contact surfaces, with the unit being millimeters.

(2) $$K=\left\{\begin{array}{c} 32.5\;\;d=0\\ 15\;\;\;\;d=0.2\\ 0\;\;\;\;\;\;\;d=1 \end{array}\right.$$

### 2.4. Determination of Rolling Process Parameters

The finishing rolling stage consists of two consecutive rolling passes. In TM-Pass 1, the workpiece sequentially passes through the Groove D-UR-C passes, while in TM-Pass 2, it sequentially traverses the Groove B-UF-A passes, thereby completing the full rolling process of the product. Numerical simulation of multi-stand continuous rolling is challenging to achieve in a single run due to high computational complexity. Therefore, single-pass rolling simulations are first performed to validate the rationality and applicability of boundary conditions. Subsequently, multi-pass continuous rolling simulations are carried out.

The process parameters of the finishing rolling process are summarized in Table 3, including the reduction, roll gap, web thickness, work roll diameter, roll rotational speed, rolling speed, and rolling force for each pass of the pass system. When the workpiece passes through the edger mill, the web thickness remains unchanged. The large deformation at the flange induces non-uniform metal flow distribution, leading to potential defects such as bending, buckling, and torsion in the workpiece. Since the steel mill did not provide the associated guide and guard devices for the F-section steel rolling process, the running stability of the workpiece was controlled by adjusting the model boundary conditions during numerical simulation. The conversion relationship between the roll angular velocity and rotational speed is given by Formula (3):

(3) $$1\;rpm=\frac{2\pi \;rad}{60\;s}\approx 0.1047\;rad/s$$

### 2.5. Finite Element Model

Given the complex cross-section of the F-section steel, to avoid excessive computational overhead and ensure the accuracy of simulation results, appropriate element types must be selected for the constructed geometric model based on the simulation category and computational requirements. The ABAQUS element library is highly comprehensive, with tetrahedral and hexahedral meshes being the most widely employed. Tetrahedral meshes are relatively easy to generate and do not require segmentation of geometric models with complex geometries. However, under the condition of identical element sizes, hexahedral meshes can reduce computational cost while achieving higher computational precision. In this study, the eight-node thermo-mechanical coupled hexahedral reduced-integration element (C3D8RT) was adopted for meshing.

**Figure 3:**
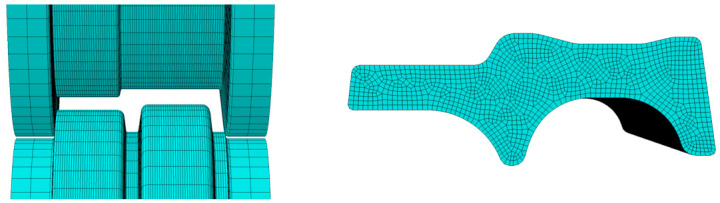
Mesh refinement region.

**Figure 4:**
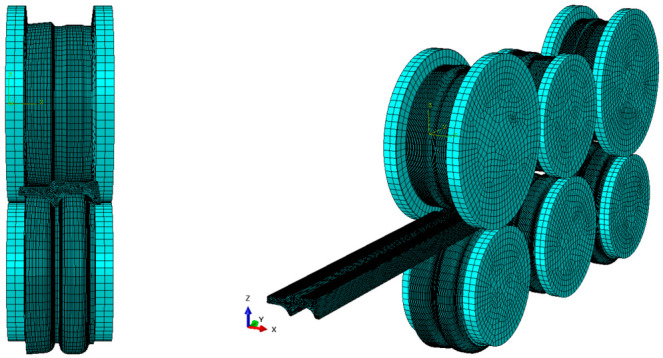
Three-dimensional finite element model of TM rolling mill.

During hot rolling, the contact interface between the workpiece and the inner surface of the roll pass undergoes significant stress and strain. To prevent solution termination caused by mesh distortion, local mesh refinement is required at the contact region. Additionally, mesh size directly influences the solution efficiency of the model: smaller mesh sizes lead to smaller time increments, thereby extending the total solution time. Thus, rational mesh sizing is essential to ensure both model convergence and computational efficiency. The mesh refinement part is shown in Figure 3, and the overall meshing of the model is shown in Figure 4.

## 3. Finite Element Simulation of F-Section Steel

**Figure 5:**
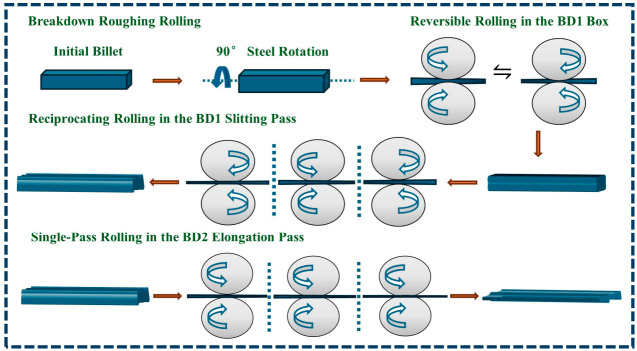
Rough rolling of the breakdown mill (BD).

The F-section steel production line at the steel plant utilizes large square billets as feedstock, with a specified cross-sectional dimension of 420 × 320 mm (width × height). To reduce computational time, a 1500 mm long billet was sectioned along the length direction to serve as the initial billet model for calculations. The on-site rolling system is composed of a breakdown (BD) roughing mill unit and tandem (TM) finishing mill unit. The BD unit is further divided into BD1 and BD2 mills, through which the billet undergoes multi-pass breakdown rolling. The TM unit consists of two universal rolling mills (UR and UF) and an intermediate edger (E), forming a three-stand continuous rolling configuration. The rough rolling process of the breakdown (BD) mill is illustrated in Figure 5.

**Figure 6:**
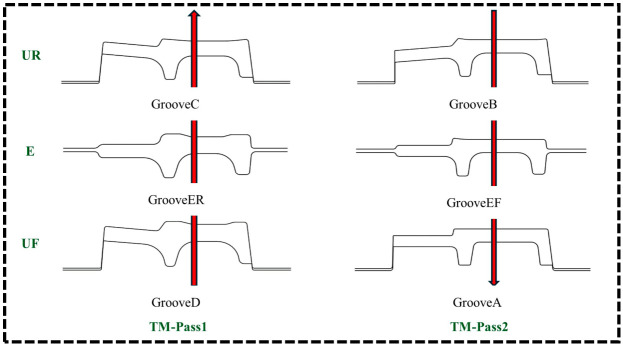
Finishing rolling of the tandem mill (TM).

The TM unit employs two continuous rolling passes: TM-Pass1 is completed via the UF-E-UR rolling sequence, while TM-Pass2 is achieved through the UR-E-UF sequence, thereby finalizing the entire rolling process of the product. During the finishing rolling process, numerical simulation of multi-stand continuous rolling is challenging to achieve in a single run. Therefore, single-pass rolling simulations are first performed to validate the rationality and applicability of boundary conditions based on the solved results, followed by the completion of multi-pass continuous rolling simulations. The finishing rolling process of the tandem mill (TM) is illustrated in the Figure 6. Herein, green text denotes different rolling mills, while red arrows indicate the rolling direction.

Based on the numerical simulation results of the finishing rolling process for F-section steel, the motion state, stress–strain distribution, temperature field, and metal flow behavior in each rolling pass were analyzed respectively. Following analysis of the available results, one roll profile was selected as the representative profile. The influence patterns of different process parameters on the pass filling degree of the rolled piece during this specific pass was further investigated.

### 3.1. Tandem Rolling State

**Figure 7:**
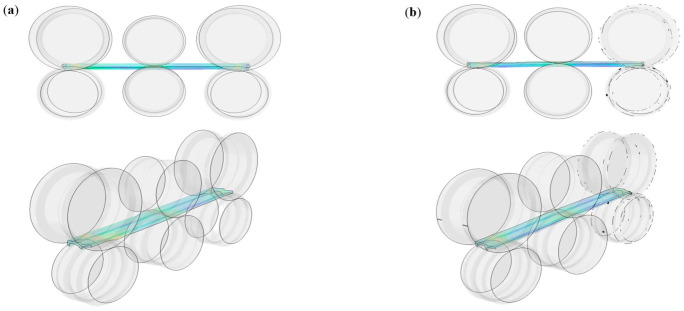
Motion state of the rolled piece in each pass. (**a**) TM-Pass 1, (**b**) TM-Pass 2.

The motion state of the rolled piece during the stable rolling stage is illustrated in Figure 7. In the two rolling passes shown in the following figure, the motion state of the rolled piece remains satisfactory, and the mass flow rate per second between each stand is basically consistent, maintaining a micro-tension rolling regime. The stacking–pulling state of the rolled piece directly determines the stable operation of the continuous rolling process. Excessive tension can cause severe steel stretching, which degrades the dimensional accuracy of the rolled piece. Conversely, insufficient tension may trigger stacking accidents, leading to interruptions in normal production.

### 3.2. Stress–Strain Contour Plot

**Figure 8:**
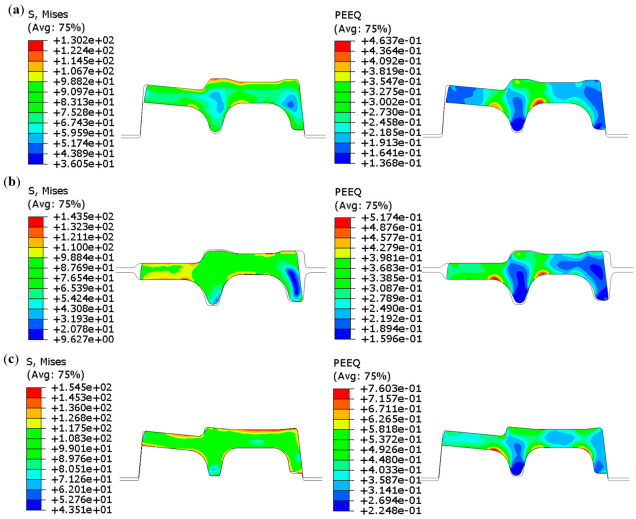
Stress–strain contour plot of the rolled piece in the TM-Pass 1. (**a**) Groove-D, (**b**) Groove-ER, (**c**) Groove-C.

**Figure 9:**
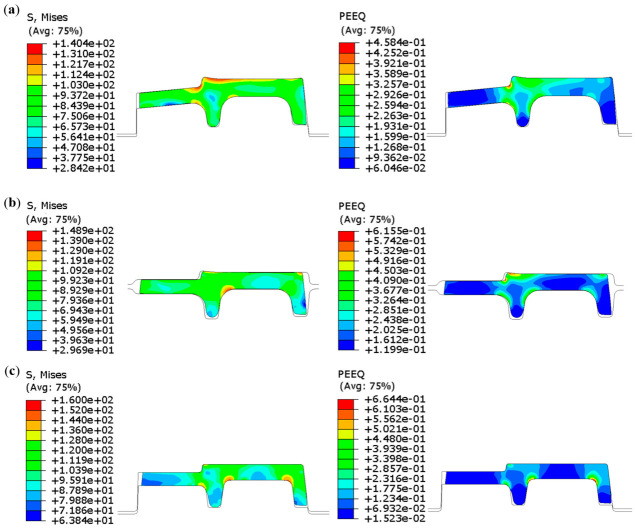
Stress–strain contour plot of the rolled piece in the TM-Pass 2. (**a**) Groove-B, (**b**) Groove-EF, (**c**) Groove-A.

The stress–strain distribution of the rolled piece during the stable rolling stage is illustrated in the Figure 8 and Figure 9. GrooveD-ER-C corresponds to the TM1 continuous rolling pass, while GrooveB-EF-A corresponds to the TM2 continuous rolling pass.

In the TM1 pass, with the accumulation of deformation of the rolled piece, the maximum stress increases from 130.2 MPa to 154.5 MPa and the maximum strain rises from 0.4637 to 0.7603. Significant deformation occurs at the junctions of the inner leg with the flange and web, whereas the deformation in the inner regions at both legs is relatively minor. Additionally, when the rolled piece passes through the Groove-C, the outer leg-web junction also exhibits higher stress–strain levels. This is attributed to narrower design of the outer leg, necessitating subsequent profile optimization.

In the TM2 pass, with the accumulation of deformation of the rolled piece, the maximum stress increases from 140.4 MPa to 160.0 MPa and the maximum strain rises from 0.4584 to 0.6644. Significant deformation occurs at the junctions of the inner leg with the flange and web, as well as at the junction of the outer leg with the web. In contrast, the inner regions of the legs and the web exhibit relatively minor deformation. When the rolled piece passes through the Groove-B, substantial deformation is observed at the flange, with a large volume of metal flowing from the flange to the web. Unbalanced metal distribution affects the exit state of the rolled piece, thereby impairing the dimensional accuracy and surface quality of the finished product. Thus, the Groove-B is selected as the representative pass profile. In the subsequent chapter, the influence of different process parameters on the pass filling degree will be investigated.

### 3.3. Pass Filling Degree

**Figure 10:**
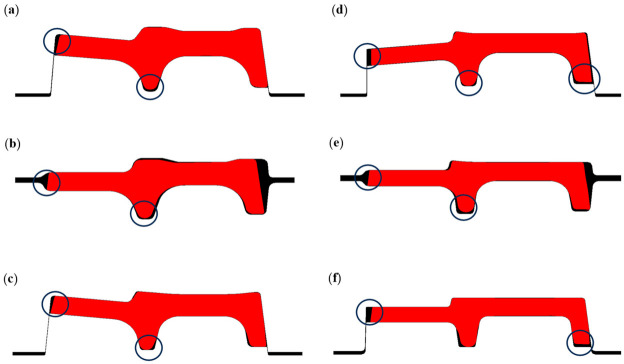
The pass filling degree of the rolled piece in each pass. (**a**) Groove-D; (**b**) Groove-ER; (**c**) Groove-C; (**d**) Groove-B; (**e**) Groove-EF; (**f**) Groove-A.

During the rolling process of special-section steel, the complexity of roll geometry renders the pass filling degree a core indicator for characterizing whether the actual product can meet the specified dimensional accuracy of the target product. Its quantification method is one of the key technical challenges in this field. This paper extracts node coordinates of the rolled piece cross-section during the stable rolling stage of each pass. These node coordinates are imported into three-dimensional modeling software to reconstruct the cross-section. The ratio of the reconstructed cross-sectional area to the theoretical pass area is defined as the pass filling degree for the corresponding pass profile. Figure 10 illustrates the filling morphology of the rolled stock at each pass during the stable rolling stage, with the unfilled regions of the rolled stock marked by circles.

Incomplete filling is observed at the flange and both leg bottoms of the rolled piece within each pass. The roll pass requires optimized design; the dimensions of the flange and the bottoms of legs should be modified based on the metal flow mechanisms. While insufficient filling is observed across all passes, the underfilled area accounted for a negligible proportion relative to the total area of pass profile. Thus, it is imperative to explore the segmentation strategy for the overall pass filling degree of the rolled piece. In this study, the pass is segmented into four regions: inner leg, outer leg, upper surface, and lower surface. The filling degree of each pass region is quantified by calculating the ratios of the rolled piece’s leg heights and surface widths to the corresponding dimensional values of the pass. The subsequent chapter will investigate the effect patterns of different process parameters on the regional pass filling degrees of the rolled piece in typical pass.

### 3.4. Validation of Simulation Results

The necessity of validating numerical simulations lies in establishing a reliable bridge between idealized mathematical models and complex physical reality, ensuring that the simulation results are not only numerically convergent but also physically authentic. By benchmarking simulation outputs against experimental observations, the validation process quantifies errors stemming from model simplifications, material property deviations, and boundary condition assumptions. This rigorous transformation elevates finite element analysis (FEA) from a mere “visual demonstration” into a scientifically persuasive and predictive tool for engineering decision making.

**Figure 11:**
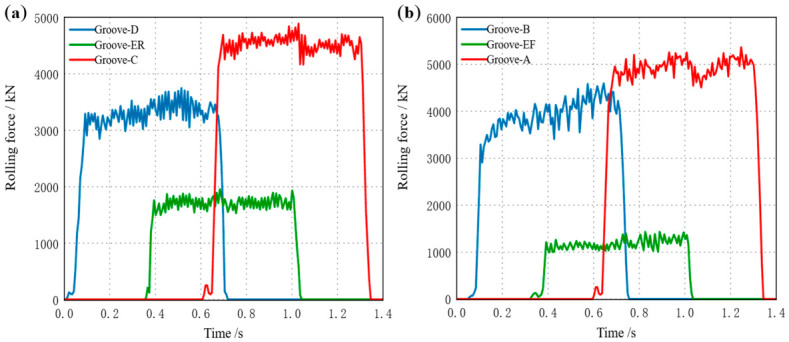
Time-variation curve of rolling force for each pass of the TM mill. (**a**) TM-Pass 1, (**b**) TM-Pass 2.

During TM1 continuous rolling, UF first bites in, followed sequentially by E and UR, establishing the continuous rolling process. During TM2 continuous rolling, UR first bites in, followed sequentially by E and UF, establishing the continuous rolling process. The stacking–drawing relationship during continuous rolling is formed based on the rolling mill speed. Observing Figure 11 reveals that whenever the workpiece is ejected from the previous roll gap, the rolling force within the subsequent roll gap fluctuates. The accuracy of the developed FE model was validated by benchmarking the steady-state rolling force simulated in ABAQUS against the measured data from the hot-rolling production line, as presented in Table 4. The results demonstrate that the relative error remains within a narrow margin of 10%. Considering the inherent complexities in industrial production—such as dynamic friction conditions, the influence of oxide scale, and sensor measurement tolerances—this level of deviation proves that the numerical model possesses high predictive fidelity in capturing high-temperature large deformation behavior and thermo-mechanical coupling effects. Thus, the model provides a reliable foundation for subsequent mechanical analysis.

**Figure 12:**
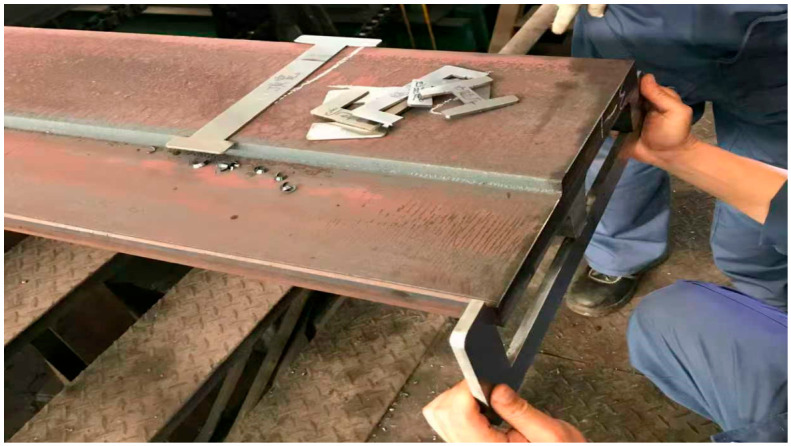
On-site dimensional measurement process of the F-section steel.

This study conducted on-site mapping and sampling of the rolled products actually produced by the steel mill. Figure 12 illustrates the practical operation process of the on-site technicians measuring the dimensions of various key parts of the rolled piece’s cross-section. To further verify the reliability of the simulation results and provide reliable data support for subsequent process optimization and theoretical analysis, this paper extracted the characteristic dimensions of the rolled piece cross-section after the TM2 pass, and compared and analyzed them with the on-site measured values. The specific data are shown in Table 5.

As shown in Table 5, the simulated values of the cross-sectional characteristic dimensions of the rolled piece after the TM2 pass are in good agreement with the on-site measured values, with the overall relative error controlled within 2%. The simulation accuracy of the thickness of the web and flange, as well as the height of the inner leg, is relatively high, with the relative error being lower than 0.3%. The height of the outer leg is affected by local under-filled defects, and the relative error with the measured values is relatively large, at 1.26%. The comparison results fully demonstrate that the predicted values of various dimensions obtained through numerical simulation have extremely high credibility and can be used as effective reference data for subsequent process analysis and theoretical analysis.

## 4. Results and Discussion

### 4.1. Effect of Friction Coefficient on Pass Filling Degree

In practical rolling, the friction coefficient dictates the stability of the initial bite. Insufficient friction causes the bite angle to exceed the friction angle, leading to bite failure. Conversely, excessive friction induces uneven stress and surface scratches, which compromises the final dimensional accuracy. Therefore, an optimal friction range is essential to ensure stable biting while preserving product quality.

**Figure 13:**
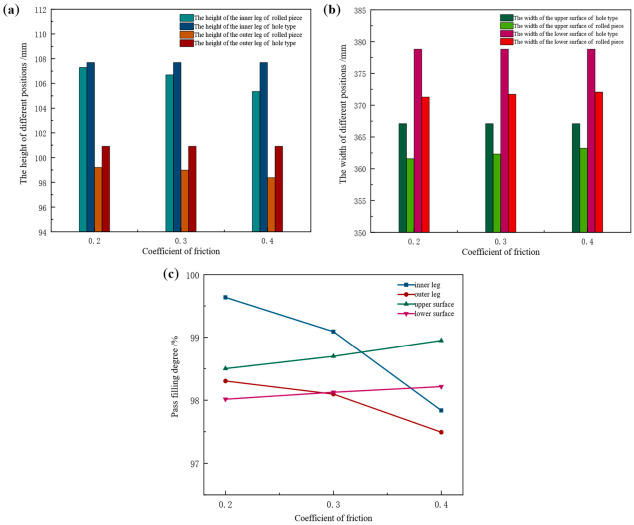
Dimension comparison between rolled piece and Groove-B at different friction coefficients. (**a**) Height values of the inner and outer legs, (**b**) width values of the upper and lower surfaces, (**c**) variation of pass filling degree.

The friction coefficient is influenced by process parameters, including contact gap size, rolling speed, and rolling temperature. Taking the Groove-B as the research object, the influence of friction coefficient on the pass filling degree of the rolled piece was investigated at constant tension configuration, rolling temperature, and web reduction. Numerical simulation of this pass was performed with friction coefficients set to 0.2, 0.3, and 0.4, respectively. The height and width values of the rolled piece during the stable rolling stage at different friction coefficients are presented in Table 4. Comparison of corresponding dimensions between the rolled piece and Groove-B pass is presented in Figure 13.

By synthesizing the data from Table 6 and the results presented in Figure 13, it can be observed that with an increase in the friction coefficient, the height of the inner leg of the rolled piece decreases from 107.29 mm to 105.35 mm, accompanied by a reduction in filling degree from 99.64% to 97.84%. The height of the outer leg decreases from 99.20 mm to 98.38 mm, with its filling degree decreasing from 98.31% to 97.49%. The width of the upper surface increases from 361.59 mm to 363.23 mm and the corresponding filling degree rises from 98.50% to 98.95%. The width of the lower surface increases from 371.29 mm to 372.05 mm, with its filling degree increasing from 98.01% to 98.22%.

Upon entering the rolling zone, the friction force acting on the rolled piece is oriented opposite to the direction of its movement and the metal experiences greater resistance during longitudinal deformation. In the height direction, increased friction hinders the reduction of the rolled piece. In accordance with the Law of Minimum Resistance, a greater volume of metal is driven to flow toward the width direction. Consequently, as the friction coefficient increases, the leg height of the rolled piece decreases while the surface width increases.

**Figure 14:**
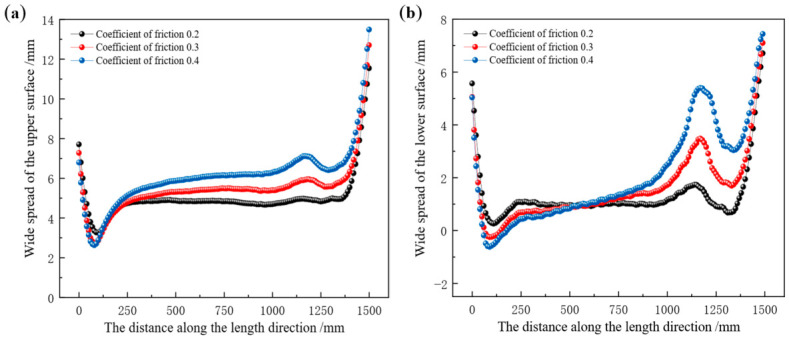
Longitudinal distribution of surface width spread of the rolled piece at different friction coefficients. (**a**) Upper surface, (**b**) lower surface.

Longitudinal distribution of surface width spread in the rolled piece at different friction coefficients is presented in Figure 14. The 0 mm position corresponds to the head of the rolled piece, while the 1500 mm position denotes the tail. The distribution of surface width spread follows a distinct pattern: the tail exhibits the maximum width spread, followed by the head, and the minimum width spread occurs at the transition zone from the head to the middle (120 mm position).

When the friction coefficient is 0.2, the width spread distribution in the longitudinal middle zone of the rolled piece remains approximately stable. As the friction coefficient increases, the distribution curve gradually steepens, with a peak emerging within the range of 1100–1200 mm. Notably, a larger friction coefficient corresponds to a higher peak position. In Figure 14a, at a friction coefficient of 0.2, the average width spread value in the longitudinal middle zone of the rolled piece is 4.84 mm. This value increases to 5.47 mm when the friction coefficient rises to 0.3 and further reaches 6.30 mm at a friction coefficient of 0.4. In Figure 14b, when the friction coefficients are 0.2, 0.3, and 0.4, the average width spread values in the middle zone are 1.04 mm, 1.41 mm, and 1.70 mm, respectively.

Under varying friction coefficients, the width spread on the upper surface of the rolled piece is consistently greater than that on the lower surface. This is attributed to the more complex contour distribution of the lower surface of the roll pass, which imposes stronger constraints on the lower surface of the rolled piece—thereby increasing the resistance to the lateral flow of the metal and resulting in a smaller width spread on the lower surface.

### 4.2. Effect of Tension Configuration on Pass Filling Degree

The presence of tension affects the deformation behavior of the rolled piece within the roll pass, thereby influencing rolling stability. During the hot-rolling process, inter-stand tension is controlled by using loopers. By contrast, during cold rolling, inter-stand tension is regulated by adjusting the roll speed between adjacent stands. In the simulation environment, different tension configurations are achieved by applying varying tensile stresses to both ends of the rolled piece.

**Figure 15:**
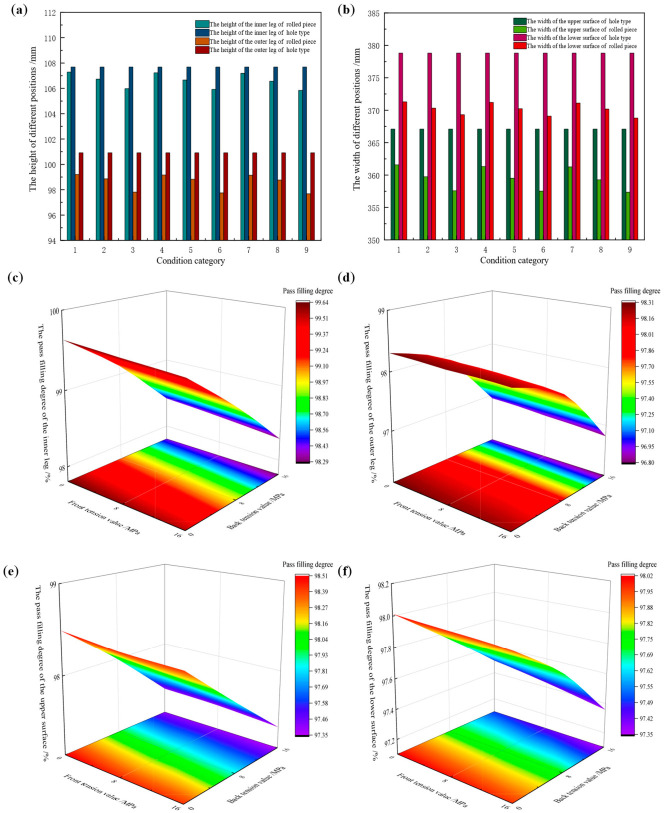
Dimension comparison between rolled piece and Groove-B at different tension configurations. (**a**) Height values of the inner and outer legs, (**b**) width values of the upper and lower surfaces, (**c**) variation in the pass filling degree of the inner leg, (**d**) variation in the pass filling degree of the outer leg, (**e**) variation in the pass filling degree of the upper surface, (**f**) variation in the pass filling degree of the lower surface.

Taking the Groove-B pass as the research object, the influence of tension configuration on the pass filling degree of the rolled piece was investigated at constant friction coefficient, rolling temperature, and web reduction. Following the single-variable principle, nine sets of condition categories were established, with each set representing a distinct tension configuration, as detailed in Table 7. Dimensional values of different regions of the rolled piece during the stable rolling stage were extracted at different tension configurations to characterize the evolution of pass filling degree. The leg height and surface width values of the rolled piece at different tension configurations are presented in Table 8. The comparison of different regions of the rolled piece and the pass is presented in Figure 15.

In Figure 15a,b, the horizontal axis represents the working conditions, indicating different tension mechanisms; the vertical axis represents the size values of different parts of the rolled piece. In Figure 15a, the legend denotes the height values of the inner and outer legs of the rolled piece and the pass; in Figure 15b, the legend denotes the width values of the upper and lower surfaces of the rolled piece and the pass. In the three-dimensional Figure 15c–f, the X-axis represents the front tension value, the Y-axis represents the back tension value, and the Z-axis represents the pass filling degree of different parts of the rolled piece. 

From Table 8 and Figure 15, it can be seen that when the same tension is applied to both ends of the rolled piece (condition categories: 1, 5, 9), as the tension value increases, the dimensions of each part of the rolled piece and pass filling degree show the following changes: the height of the inner leg decreases from 107.29 mm to 105.84 mm, accompanied by a reduction in the pass filling degree from 99.64% to 98.29%. The height of the outer leg decreases from 99.20 mm to 97.68 mm, with the pass filling degree dropping from 98.31% to 96.80%. Meanwhile, the width of the upper surface decreases from 361.59 mm to 357.35 mm and the pass filling degree decreases from 98.50% to 97.35%. The width of the lower surface decreases from 371.29 mm to 368.79 mm, with the pass filling degree declining from 98.01% to 97.35%.

When different tensions are applied at both ends of the rolled piece, with the front tension remaining constant at 8 MPa (condition categories: 4, 5, 6), as the value of the back tension increases, the trend of changes in the leg height and surface width of the rolled piece is consistent: the height of the inner leg decreases from 107.23 mm to 105.92 mm, accompanied by a reduction in the pass filling degree from 99.58% to 98.37%. The height of the outer leg decreases from 99.16 mm to 97.75 mm, with the pass filling degree dropping from 98.27% to 96.87%. Similarly, the upper surface width decreases from 361.33 mm to 357.53 mm and its pass filling degree decreases from 98.43% to 97.40%. The lower surface width decreases from 371.20 mm to 369.08 mm, with the pass filling degree declining from 97.99% to 97.43%.

When the back tension is held constant at 8 MPa (condition categories: 2, 5, 8), as the front tension increases, the changes in the dimensions of each part and pass filling degree are minimal. The height of the inner leg decreases from 106.73 mm to 106.56 mm, with the pass filling degree reducing from 99.12% to 98.96%. The height of the outer leg decreases from 98.86 mm to 98.76 mm, with the pass filling degree declining from 97.97% to 97.87%. The upper surface width decreases from 359.74 mm to 359.26 mm, accompanied by a pass filling degree that falls from 98.00% to 97.87%. The lower surface width decreases from 370.32 mm to 370.18 mm, with the pass filling degree decreasing from 97.76% to 97.72%.

By synthesizing variations in the leg height and surface width of the rolled piece during the stable rolling stage at different tension configurations, it can be observed that when the front and back tensions are equal, or when the front tension is held constant while only the back tension is adjusted, both the leg height and surface width of the rolled piece decrease significantly with increasing tension magnitude. In contrast, when the back tension is fixed and only the front tension is varied, the reduction in leg height and surface width is negligible. This finding demonstrates that the pass filling degree of the rolled piece at both the legs and surfaces is predominantly governed by the back tension. Subsequent studies will focus on the longitudinal spread distribution characteristics of the rolled piece surface under different front–back tension configurations. 

**Figure 16:**
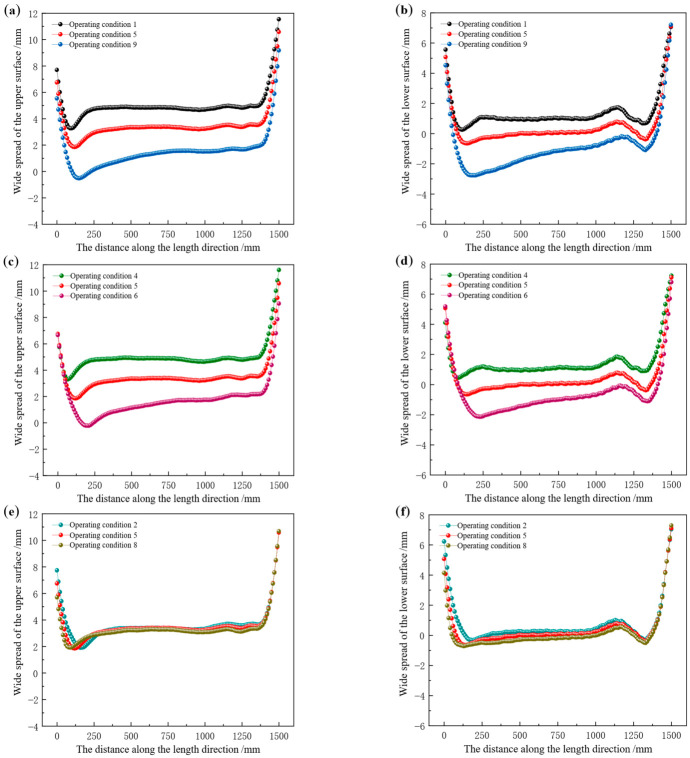
Longitudinal distribution of surface width spread of the rolled piece at different tension configurations. (**a**) The upper surface subjected to identical front and back tensions, (**b**) the lower surface subjected to identical front and back tensions, (**c**) the upper surface with a fixed front tension of 8 MPa while varying the back tension, (**d**) the lower surface with a fixed front tension of 8 MPa while varying the back tension, (**e**) the upper surface with a fixed back tension of 8 MPa while varying the front tension, (**f**) the lower surface with a fixed back tension of 8 MPa while varying the front tension.

The width spread distribution along the length direction of the rolled piece surface after rolling under different tension configurations is presented in Figure 16. When the front and back tensions are equal, or the front tension is constantly set at 8 MPa while only adjusting the back tension, the distribution of width spread on the surface of the rolled piece shows a consistent trend of change. As the tension increases, the distribution curve of width spread becomes increasingly unstable. In Figure 16a, as the tension value increases, the average width spread on the upper surface of the rolled piece decreases from 4.84 mm to 1.45 mm; in Figure 16c, as the back tension value increases, the average width spread on the upper surface of the rolled piece decreases from 4.78 mm to 1.54 mm; in Figure 16b, as the tension value increases, the average width spread on the lower surface of the rolled piece decreases from 1.04 mm to −1.16 mm; in Figure 16d, as the back tension value increases, the average width spread on the lower surface of the rolled piece decreases from 0.99 mm to −1.05 mm.

When the back tension is constant at 8 MPa while the front tension varies, the changes in the surface widening of the rolled piece under different tension mechanisms are relatively small. In Figure 16e, as the value of the front tension increases, the average width spread on the upper surface of the rolled piece decreases from 3.34 mm to 3.12 mm; in Figure 16f, as the value of the front tension increases, the average width spread on the lower surface of the rolled piece decreases from 0.18 mm to −0.08 mm. Based on the above data analysis, it can be concluded that either increasing the front tension alone or the back tension alone will result in a reduction in the surface width spread of the rolled piece. 

Under different tension configurations, the width spread on the upper surface of the rolled piece is consistently greater than that on the lower surface. As tension increases, the width spread on the lower surface may even become negative, which is attributed to the fact that tension induces far greater metal flow in the longitudinal direction than in the transverse direction. When front and back tensions are identical, or when the front tension is fixed at 8 MPa while the back tension is varied, increasing tension leads to a significant reduction in width spread, accompanied by a steeper distribution curve and an overall shift along the longitudinal direction—whereby the curve’s minima and maxima occur near the tail of the rolled piece. When the back tension is fixed at 8 MPa while the front tension is varied, the reduction amplitude of width spread is negligible, and the distribution curve shifts overall along the negative longitudinal direction, with minima and maxima appearing near the head of the rolled piece.

Through analysis, the following conclusion can be drawn: under different tension configurations, whether it is the metal flow in the legs of the rolled piece or the metal flow on the surface, back tension plays a decisive role. This provides theoretical guidance and data support for avoiding the formation of excessive defects and folds in the production of special-section steel.

### 4.3. Effect of Rolling Temperature on Pass Filling Degree

**Figure 17:**
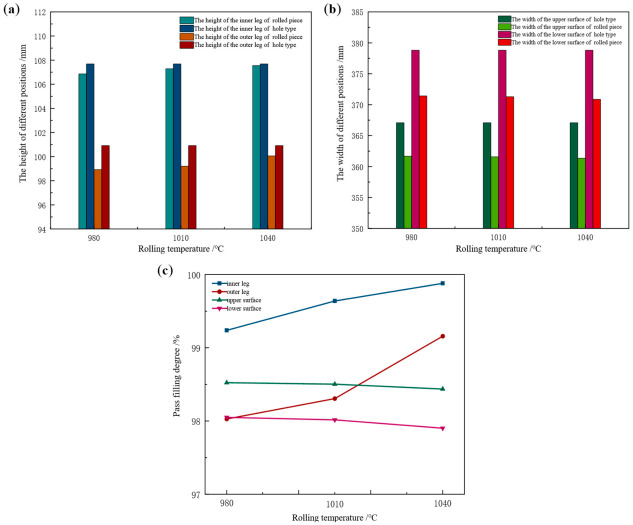
Dimension comparison between rolled piece and Groove-B at different rolling temperatures. (**a**) Height values of the inner and outer legs, (**b**) width values of the upper and lower surfaces, (**c**) variation of pass filling degree.

Taking the Groove-B pass as the research object, the influence of rolling temperature on the pass filling degree of the rolled piece was investigated at constant friction coefficient, tension configuration, and web reduction. The temperature of the rolled piece upon furnace exit is 1200 °C. After the breakdown rolling stage, the temperature is approximately 1050 °C. Prior to the initiation of the finishing rolling stage, the temperature is maintained at no less than 1040 °C. Following the completion of finishing rolling, the temperature is around 950 °C. Accordingly, rolling temperatures of 980 °C, 1010 °C, and 1040 °C were selected to perform the rolling process simulation for this pass. The height and width values of the rolled piece during the stable rolling stage at different rolling temperatures are presented in Table 9. Comparison of corresponding dimensions between rolled piece and Groove-B pass is presented in Figure 17.

From Table 9 and Figure 17, it can be seen that the increase in rolling temperature has opposite effects on the pass filling degree of the leg and surface of the rolled piece. In the vertical direction, the height of the inner leg of the rolled piece increases from 106.86 mm to 107.55 mm, accompanied by an increase in filling degree from 99.24% to 99.88%. The height of the outer leg increases from 98.92 mm to 100.06 mm, with the filling degree rising from 98.03% to 99.16%. In the width direction, the width of the upper surface decreases from 361.67 mm to 361.35 mm, corresponding to a filling degree that decreases from 98.52% to 98.44%. The width of the lower surface decreases from 371.42 mm to 370.86 mm, with the filling degree showing a similar trend of decreasing from 98.05% to 97.90%.

This behavior is ascribable to the significant reduction in the material’s yield strength and deformation resistance with increasing rolling temperature. The metal softens, its flowability is enhanced, and plastic deformation is facilitated, thereby promoting an increase in the leg height of the rolled piece. Rolling temperature primarily modulates the friction coefficient by altering the properties of the iron oxide scale, thus exerting an indirect influence on width spread. During the finishing rolling stage, the melting of the iron oxide scale imparts a lubricating effect, lowering the friction coefficient and consequently reducing width spread.

**Figure 18:**
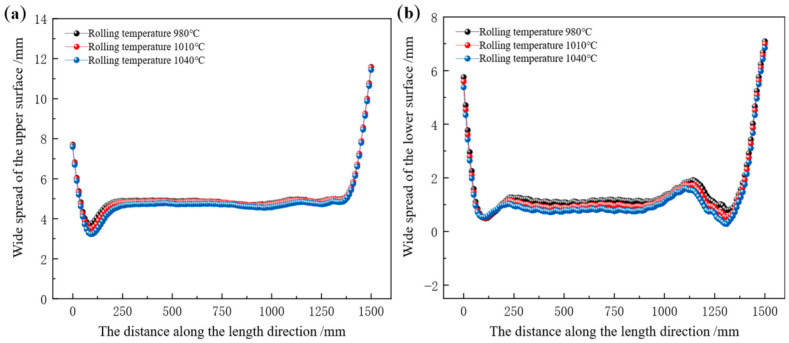
Longitudinal distribution of surface width spread of the rolled piece at different rolling temperatures. (**a**) Upper surface, (**b**) lower surface.

Longitudinal distribution of surface width spread in the rolled piece at different rolling temperatures is presented in Figure 18. With increasing rolling temperature, the width spread distribution remains stable with negligible variation. In Figure 18a, when rolling temperatures are 980 °C, 1010 °C, and 1040 °C, respectively, the average spread in the longitudinal middle region of the rolled piece is 4.92 mm, 4.84 mm, and 4.65 mm in sequence. In Figure 18b, a distinct peak is observed in the range of 1100–1200 mm. When rolling temperatures are 980 °C, 1010 °C, and 1040 °C, respectively, the average spread is 1.20 mm, 1.04 mm, and 0.72 mm in sequence.

For profiled steel, the heat dissipation surface area per unit volume varies drastically between regions of different thicknesses, leading to significant cross-sectional temperature differences during rolling [26]. The non-uniform temperature field distribution gives rise to varying deformation resistances across the cross-section, leaving substantial residual stresses in the workpiece after it exits the rolls and ultimately causing severe forming defects. Therefore, the rational control of rolling temperature is of critical importance.

### 4.4. Effect of Web Reduction on Pass Filling Degree

The rational allocation of reduction is the core of the continuous rolling process for section steel. It not only regulates metal flow to ensure the cross-sectional profile and pass filling degree, but also balances the mill load and suppresses forming defects by optimizing the internal stress state.

In the multi-pass continuous rolling process, the absolute reduction between successive passes typically exhibits a gradually decreasing trend. During the roughing stage, the rolled piece achieves large deformation through substantial reduction; by the end of roughing, the cross-section of the rolled piece has basically formed the required profiled contour. In the finishing stage, however, as the thickness of the rolled piece has been reduced, small and precise reductions must be employed to strictly control the dimensional accuracy and surface quality of the final product, thereby avoiding side bending and torsion defects caused by excessive reduction.

In the Groove-B rolling pass, the web thickness of the rolled piece is 41.7 mm, the web thickness of the pass is 37.7 mm, and the reduction is 4 mm. Different reductions are achieved by adjusting the roll gap between the upper and lower rolls. Since the web thickness of the Groove-A pass in the final pass is 36.5 mm, to ensure rolling stability, three sets of different web thicknesses are set in accordance with the principle of gradual reduction, as shown in Table 10.

**Figure 19:**
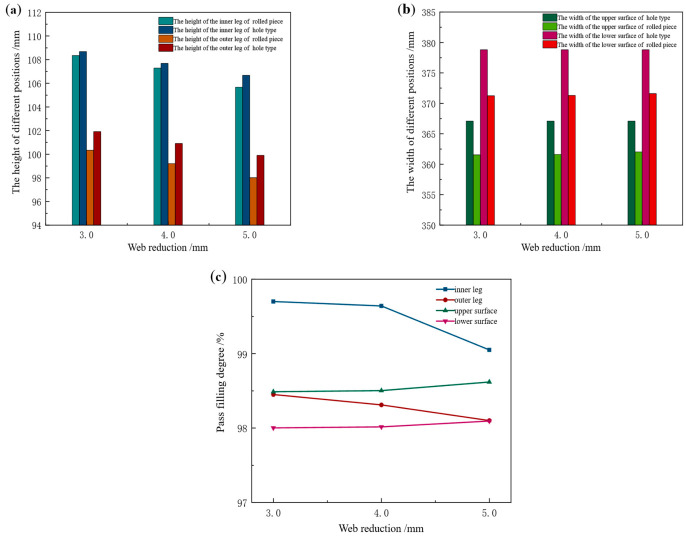
Dimension comparison between rolled piece and Groove-B under different web reduction conditions. (**a**) Height values of the inner and outer legs, (**b**) width values of the upper and lower surfaces, (**c**) variation of pass filling degree.

Taking the Groove-B pass as the research object, the influence of web reduction on the pass filling degree of the rolled piece was investigated at constant friction coefficient, rolling temperature, and tension configuration. In each working condition, the pass height varies with the setting of web reduction, while the pass width remains constant. By extracting the cross-sectional dimensions of key positions of the rolled piece during the stable rolling stage and comparing them with the corresponding pass design dimensions, the pass filling degree is quantified. The leg height and surface width values of the rolled piece during the stable rolling stage under different web reduction conditions are presented in Table 11 and Table 12, respectively. The comparison of dimensional values between the rolled piece and the pass, as well as the variation of filling degree during the stable rolling stage, is illustrated in Figure 19.

Based on Table 11 and Table 12, and Figure 19, it can be concluded that an increase in web reduction exerts diametrically opposite effects on the pass filling degree of the rolled piece’s legs and surfaces. Specifically, in the height direction: the inner leg height of the rolled piece decreases from 108.35 mm to 105.67 mm, with the corresponding pass filling degree reducing from 99.70% to 99.05%. The outer leg height decreases from 100.33 mm to 98.01 mm, and the pass filling degree decreases from 98.45% to 98.10%. In the width direction: the upper surface width of the rolled piece increases from 361.54 mm to 362.02 mm, with the corresponding pass filling degree rising from 98.49% to 98.62%. The lower surface width increases from 371.24 mm to 371.59 mm, and the pass filling degree increases from 98.00% to 98.09%.

This is attributed to the fact that an increase in web reduction leads to an extension of the deformation zone length of the rolled piece and an expansion of the horizontal projection shape of the deformation zone, thereby increasing the resistance to longitudinal plastic flow and the magnitude of the longitudinal compressive principal stress. In the height direction, since grooves are provided at the bottom of the inner and outer legs of the pass, the metal is required to travel a longer distance to reach the bottom of the inner and outer legs. Simultaneously, the side walls of the grooves exert normal squeezing force and tangential friction force on the metal, and this strong geometric constraint results in a more significant cumulative effect of frictional resistance. According to the law of minimum resistance, the metal preferentially flows in the lateral direction. Consequently, as the web reduction increases, the height of the inner and outer legs of the rolled piece decreases, while the width of the upper and lower surfaces increases.

**Figure 20:**
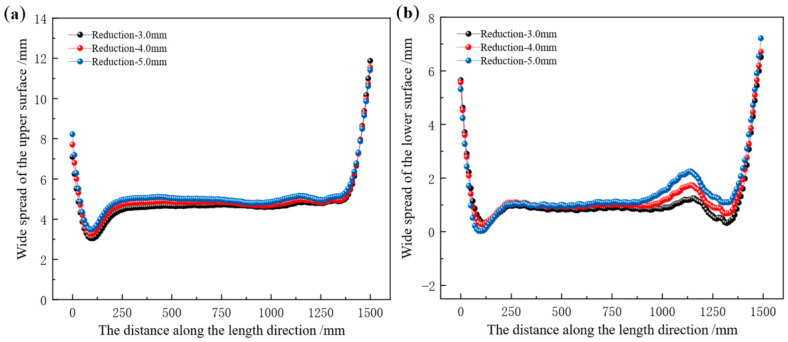
Longitudinal distribution of surface width spread of the rolled piece under different web reduction conditions. (**a**) Upper surface, (**b**) lower surface.

Figure 20 presents the longitudinal distribution curves of the surface spread of the rolled piece under different web reduction conditions. In Figure 20a, the surface spread distribution in the longitudinal middle region of the rolled piece is approximately stable. When web reductions are 3 mm, 4 mm, and 5 mm, respectively, the average spread in the longitudinal middle region of the rolled piece is 4.74 mm, 4.84 mm, and 5.23 mm in sequence.

In Figure 20b, with the increase of web reduction, the spread distribution curve becomes increasingly steep, and a peak emerges in the range of 1100 mm–1200 mm. The larger the reduction, the higher the peak. When web reductions are 3 mm, 4 mm, and 5 mm respectively, the average spread is 0.99 mm, 1.04 mm, and 1.25 mm in sequence.

An increase in web reduction not only increases the surface spread of the rolled piece but also alters its overall spread distribution. The spread on the upper surface of the rolled piece is consistently greater than that on the lower surface. This is because the profile distribution of the lower roll is more complex, which exerts a stronger constraint on the lower surface of the rolled piece, thereby increasing the resistance to the lateral flow of metal. According to the law of minimum resistance, metal preferentially flows in the direction of least resistance, more metal flows toward the upper surface—ultimately resulting in a smaller spread on the lower surface.

## 5. Conclusions

In this study, combined with the rolling schedule of the steel plant production line, the ABAQUS 2022 finite element simulation software was employed to carry out a simulation investigation on the finishing rolling process of F-section steel. The deformation laws of the rolled piece were systematically summarized, and the effects of friction coefficient, tension mechanism, rolling temperature, and web reduction on pass filling degree were analyzed in depth. The specific results are as follows:

(1) During finishing rolling, the maximum deformation of the rolled piece is concentrated at the junctions of the inner leg with the flange, the inner leg with the web, and the outer leg with the web. The underfilling phenomenon is observed in the flange and legs of each pass. The relative errors between the rolling force and the characteristic dimensions of the rolled piece cross-section obtained via numerical simulation and the on-site actual values were controlled within 10% and 2%, respectively, which verifies the accuracy of the simulation results.
(2) Conditions of low friction, small reduction, and high temperature facilitate the smooth filling of metal in the leg cavity; in contrast, conditions of high friction, large reduction, and low temperature promote the filling of surface metal and an increase in spread. Maintaining a low-tension state is a common favorable condition for improving the pass filling degree of both the legs and the surface.
(3) From an industrial standpoint, the implementation of an effective lubrication mechanism can reduce friction, lower rolling loads, and facilitate rolling reduction. The application of appropriate tension can prevent the formation of overfilling defects and folds. By optimizing the reduction schedule and rationally distributing metal elongation across different cross-sectional regions, the deformation concentration within the pass can be effectively mitigated. The rational control of rolling temperature is not only associated with the flow and filling behavior of metal within the pass, but also serves as a critical measure to prevent surface cracks and folding defects.

## Figures and Tables

**Table 1:** Material properties of Q235B carbon steel.

Temperature (°C)	800	900	1000	1100	1200
Young’s Modulus (MPa)	1.27 × 10^5^	1.17 × 10^5^	1.06 × 10^5^	0.96 × 10^5^	0.86 × 10^5^
Poisson’s Ratio	0.33	0.35	0.35	0.36	0.36
Density (g/cm^3^)	7.61	7.60	7.54	7.49	7.44
Expansion Coefficient	1.48 × 10^−5^	1.54 × 10^−5^	1.39 × 10^−5^	1.37 × 10^−5^	1.35 × 10^−5^
Thermal Conductivity (mW/(mm.°C))	25.98	26.58	27.18	28.43	29.68
Specific Heat (mJ/(tone.°C))	9.59 × 10^8^	6.09 × 10^8^	6.25 × 10^8^	6.40 × 10^8^	6.57 × 10^8^

**Table 2:** Thermal boundary condition parameters.

Thermal Boundary Conditions	Parameter
Film coefficient (mW/(mm^2^·°C))	0.065
Emissivity	0.8
Ambient temperature (°C)	20
Roll temperature (°C)	200
Initial temperature of rolled piece (°C)	1200
Plastic Work Heat Generation Coefficient	0.9
Friction Heat Generation Coefficient	0.5

**Table 3:** Rolling process parameters of the finishing mill.

Pass Profile	Reduction (mm)	Thickness (mm)	Roll Gap (mm)	Roll Speed Rpm (1/min)	Rolling Speed (m/s)	Rolling Force (ton)
Groove-D	7	47.7	5.5	64.6	3.22	322
Groove-ER	0	47.7	10	69.7	—	188
Groove-C	6	41.7	5.5	74.2	3.98	457
Groove-B	4	37.7	5.5	78.9	4.00	412
Groove-EF	0	37.7	10	81.7	—	121
Groove-A	1.2	36.5	5.5	83.1	4.47	518

**Table 4:** Steady-state rolling force and error in the finishing mill.

Pass Profile	Simulated Value/kN	Measured Value/kN	Error Value/KN	Error Rate/%
Groove-D	3432	3158	274	7.98%
Groove-ER	1789	1843	54	3.02%
Groove-C	4703	4482	221	4.93%
Groove-B	4197	4040	157	3.89%
Groove-EF	1205	1187	18	1.49%
Groove-A	4935	5080	145	2.85%

**Table 5:** Comparison of simulated and measured cross-sectional dimensions after TM2 pass.

Measurement	Simulated Value/mm	Measured Value/mm	Relative Error/%
Web thickness	36.42	36.5	0.22
Flange thickness	30.47	30.5	0.10
Inner leg height	100.21	100.3	0.09
Outer leg height	99.04	100.3	1.26
Upper surface width	366.51	368.5	0.54
Lower surface width	376.45	378.0	0.41

**Table 6:** Dimensional values of the rolled piece during the stable rolling stage at different friction coefficients.

	Inner Leg Height /mm	Outer Leg Height /mm	Upper Surface Width /mm	Lower Surface Width /mm
Groove-B pass	107.68	100.91	367.09	378.81
Friction coefficient-0.2	107.29	99.20	361.59	371.29
Friction coefficient-0.3	106.70	98.99	362.31	371.71
Friction coefficient-0.4	105.35	98.38	363.23	372.05

**Table 7:** Tension configurations at different condition categories.

Condition Category	Front Tension/MPa	Back Tension/MPa
1	0	0
2	0	8
3	0	16
4	8	0
5	8	8
6	8	16
7	16	0
8	16	8
9	16	16

**Table 8:** Dimensional values of the rolled piece during the stable rolling stage at different condition categories.

Condition Category	Inner Leg Height /mm	Outer Leg Height /mm	Upper Surface Width /mm	Lower Surface Width /mm
Groove-B	107.68	100.91	367.09	378.81
1	107.29	99.20	361.59	371.29
2	106.73	98.86	359.74	370.32
3	105.98	97.81	357.58	369.31
4	107.23	99.16	361.33	371.20
5	106.65	98.83	359.51	370.24
6	105.92	97.75	357.53	369.08
7	107.18	99.14	361.27	371.10
8	106.56	98.76	359.26	370.18
9	105.84	97.68	357.35	368.79

**Table 9:** Dimensional values of the rolled piece during the stable rolling stage at different rolling temperatures.

	Inner Leg Height /mm	Outer Leg Height /mm	Upper Surface Width /mm	Lower Surface Width /mm
Groove-B pass	107.68	100.91	367.09	378.81
Rolling temperature-980 °C	106.86	98.92	361.67	371.42
Rolling temperature-1010 °C	107.29	99.20	361.59	371.29
Rolling temperature-1040 °C	107.55	100.06	361.35	370.86

**Table 10:** Web reduction under different operating conditions.

Condition Category	Web Thickness of Rolled Piece/mm	Web Thickness of the Pass/mm	Web Reduction/mm	Web Reduction Rate/%
1	41.7	38.7	3	7.19
2	41.7	37.7	4	9.59
3	41.7	36.7	5	12.00

**Table 11:** Leg height values of the rolled piece and roll pass during the stable rolling stage under different web reduction conditions.

Web Reduction /mm	Inner Leg Height of Rolled Piece/mm	Inner Leg Height of the Pass/mm	Outer Leg Height of Rolled Piece/mm	Outer Leg Height of the Pass/mm
3	108.35	108.68	100.33	101.91
4	107.29	107.68	99.20	100.91
5	105.67	106.68	98.01	99.91

**Table 12:** Surface width values of the rolled piece and roll pass during the stable rolling stage under different web reduction conditions.

Web Reduction /mm	Upper Surface Width of Rolled Piece/mm	Upper Surface Width of the Pass/mm	Lower Surface Width of Rolled Piece/mm	Lower Surface Width of the Pass/mm
3	361.54	367.09	371.24	378.81
4	361.59	367.09	371.29	378.81
5	362.02	367.09	371.59	378.81

## Data Availability

The original contributions presented in this study are included in the article. Further inquiries can be directed to the corresponding author.
